# Voluntary Exercise Improves Cardiac Function and Prevents Cardiac Remodeling in a Mouse Model of Dilated Cardiomyopathy

**DOI:** 10.3389/fphys.2017.00899

**Published:** 2017-11-15

**Authors:** Robin Deloux, Damien Vitiello, Nathalie Mougenot, Philippe Noirez, Zhenlin Li, Mathias Mericskay, Arnaud Ferry, Onnik Agbulut

**Affiliations:** ^1^Sorbonne Universités, UPMC University Paris 06, Institut de Biologie Paris-Seine, UMR Centre National de la Recherche Scientifique 8256, Biological Adaptation and Aging, Paris, France; ^2^UMR-S 1180, National Institute for Health and Medical Research, University Paris-Sud, Université Paris-Saclay, Châtenay-Malabry, France; ^3^Sorbonne Paris Cité, Université Paris Descartes, Paris, France; ^4^Institute for Research in Medicine and Epidemiology of Sport, EA7329, National Institute of Sport, Expertise and Performance, Université Paris Descartes, Paris, France; ^5^Sorbonne Universités, UPMC University Paris 06, UMS28, Plateforme d'Expérimentation Coeur, Muscles, Vaisseaux, Paris, France; ^6^Sorbonne Universités, UPMC University Paris 06, Institut de Myologie, UMR-S 794, National Institute for Health and Medical Research, UMR Centre National De La Recherche Scientifique 7215, Paris, France

**Keywords:** non-ischemic cardiac disease, dilated cardiomyopathy, non-forced exercise, wheel exercise

## Abstract

**Objective:** Despite the indubitable beneficial effect of exercise to prevent of cardiovascular diseases, there is still a lack of studies investigating the impact of exercise in non-ischemic dilated cardiomyopathy. Here, we investigated the impact of voluntary exercise on cardiac function in a mouse model of non-ischemic dilated cardiomyopathy (αMHC-MerCreMer:Sf/Sf), induced by cardiac-specific inactivation of the Serum Response Factor.

**Materials and Methods:** Seven days after tamoxifen injection, 20 αMHC-MerCreMer:Sf/Sf mice were assigned to sedentary (*n* = 8) and exercise (*n* = 12) groups. Seven additional αMHC-MerCreMer:Sf/Sf mice without tamoxifen injection were used as control. The exercise group performed 4 weeks of voluntary running on wheel (1.8 ± 0.12 km/day). Cardiac function, myocardial fibrosis, and mitochondrial energetic pathways were then blindly assessed.

**Results:** Exercised mice exhibited a smaller decrease of left ventricular (LV) fractional shortening and ejection fraction compared to control mice. This was associated with a lower degree of LV remodeling in exercised mice, as shown by a lower LV end-systolic intrerventricular septal and posterior wall thickness decrease from baseline values compared to sedentary mice. Moreover, exercised mice displayed a reduced gene expression of atrial and brain natriuretic factors. These benefits were associated by a reduced level of myocardial fibrosis. In addition, exercised mice exhibited a higher mitochondrial aconitase, voltage-dependent anion-selective channel 1 and PPAR gamma coactivators-1 alpha proteins levels suggesting that the increase of mitochondrial biogenesis and/or metabolism slowed the progression of dilated cardiomyopathy in exercised animals.

**Conclusions:** In conclusion, our results support the role of voluntary exercise to improve outcomes in non-ischemic dilated heart failure (HF) and also support its potential for a routine clinical use in the future.

## Introduction

Heart failure (HF) is the common final stage of most cardiovascular disorders and despite significant advances in drug management and interventional procedures, it remains one of the highest causes of morbidity and mortality worldwide (Rodriguez et al., 2013). HF is the final consequence of a complex cardiac remodeling initiated by different types of hemodynamic stress, such as myocardial infarction, chronic arterial hypertension, or fibrosis. Cardiomyopathies are the leading causes of HF in the world (Stehlik et al., 2012). Among them, dilated cardiomyopathy (DCM) represents the most frequent disease leading to HF in young adult patients (Stehlik et al., 2012; Savvatis et al., 2015). However, the molecular mechanisms leading to DCM (and lately HF) can be very heterogeneous. The DCM is a very heterogeneous pathology and involves many perturbations of the protein homeostasis in sarcomeres, nuclear membrane and cytoskeleton, or calcium metabolism. In the cytoskeleton, actin, and myosin are particularly affected (Fatkin and Graham, 2002). Despite the improvement of medication management and interventional procedures, the treatment of DCM remains to be addressed. This is due to the fact that in contrast to the ischemic forms of HF which can now be treated with procedures such as revascularization, valve repair, or remodeling operations, the only option for DCM treatment, once the disease has reached a terminal stage of drug refractoriness remains heart transplantation (Hershberger et al., 2010). In this setting, less invasive therapeutic and/or preventive approaches like physical activity, would signify a promising non-pharmaceutical, preventive therapeutic strategy for this category of HF patients.

Exercise training has emerged as an alternative non-pharmaceutical strategy to prevent cardiovascular diseases and current evidence has pointed out the usefulness of exercise training for secondary prevention of HF (Sharma et al., 2001; Guyatt and Devereaux, 2004; Downing and Balady, 2011; Gasiorowski and Dutkiewicz, 2013; Gayda et al., 2016; Ribeiro et al., 2017). Despite that exercise training yields an improvement of cardiac function and quality of life in HF patients, its effects on humans are still controversial. In the case of chronic HF, it has been shown that long-term exercise at a moderate intensity decreases mortality and hospital readmission for HF and increases the quality of life after a cardiac infarction (Belardinelli et al., 1999). In addition, a significant, but modest effect of exercise training on reduction of the combined end-point was reported by the largest trial of exercise training in chronic HF (O'Connor et al., 2009). However, other studies have reported that HF with preserved LV ejection fraction (HFpEF) was associated with a decline of cardiovascular reserve leading to exercise intolerance, particularly when it has been difficult to stabilize the heart rate after a short exercise (Upadhya et al., 2015; Gupte and Hamilton, 2016). These discrepancies regarding the benefits of chronic exercise could be explained by the variability of exercise training intensity levels and duration or by the age of patients and the different physiopathology in HFpEF compared to HF with reduced LV ejection fraction (HFrEF) that were mostly recruited in the other studies (Belardinelli et al., 1999; O'Connor et al., 2009). The molecular and cellular mechanisms involved in the insufficient heart with physical activity are still poorly known. In this study, we aimed to determine the impact of voluntary exercise during the establishment of HF in a mouse model of DCM. For this purpose, we selected a mouse model of non-ischemic DCM triggered by cardiac-specific inducible inactivation of the Serum Response Factor (SRF-HKO mice) (Parlakian et al., 2005; Diguet et al., 2011), a major regulator of cardiac genes and microRNAs that is repressed in human failing hearts (Davis et al., 2002; Chang et al., 2003). Results of the current work strongly supported the role of voluntary exercise to improve outcomes in non-ischemic HF and support its potential for a clinical use in the future.

## Materials and methods

### Animals and voluntary exercise

All procedures were performed in accordance with national and European legislations, in conformity with the Public Health Service Policy on Human Care and Use of Laboratory Animals, and were approved by our institutional Ethics Committee “Charles Darwin” (Permit number: #4370). In this study, 27 nine-month old conditionally invalidated serum response factor (SRF) mice (αMHC-MerCreMer:Sf/Sf) were used. The Cre-mediated excision of floxed SRF alleles in the heart was induced by daily intraperitoneal tamoxifen (20 mg/kg/day; Sigma-Aldrich, Saint-Quentin Fallavier, France) injections on three consecutive days. Seven days after first tamoxifen injection, αMHC-MerCreMer:Sf/Sf mice were allocated to sedentary (SRF-HKO, *n* = 8) and exercise (SRF-HKO wheel, *n* = 12) groups. Moreover, seven αMHC-MerCreMer:Sf/Sf mice without tamoxifen treatment were used as control (sedentary). Each voluntary active mouse was housed in an individual cage containing a wheel during 4 weeks, after 7 days of acclimation to the running wheel. It should be noted that mice of the exercise group were selected based on their capacity (i.e., running distance achieved) to run into wheel. The wheels were equipped with a magnet mounted on an outside surface and magnetic sensor connected with a digital counter and timer. Thus, an accurate measurement of the daily running distance and running duration was performed for each mouse in each of the 4 weeks of the voluntary training protocol. To reproduce the same experimental condition sedentary animals were also housed in an individual cage and were fed with standard chow diet containing 16% protein, 3% lipids (A04-10, Safe Scientific Animal Food & Engineering, Augy, France).

### Echocardiography

Echocardiography was performed on anesthetized mice under isoflurane (induction with 2% and maintained with 0.5%; Ferry et al., 2015). Non-invasive measurements of left ventricular (LV) dimensions were done using echocardiography-Doppler (Vivid 7 Dimension/Vivid7 PRO; GE Medical System Co, Vélizy, France) with an ultrasound probe at 9–14 MHz frequency range. The bi-dimensionally guided time-motion recording mode (parasternal long-axis view) of the left ventricle (LV) provided the following measurements: diastolic (IVSd) and systolic intrerventricular septal (IVSs) and posterior wall thicknesses (LVPWd and LVPWs), LV end-diastolic (LVEDD) and end-systolic diameters (LVESD) and heart rate. Each set of measurements was obtained from the same cardiac cycle. LV shortening fraction (LVSF) was calculated using the formula: (LVEDD-LVESD)/LVEDD × 100. LV myocardial volume (LVV), LV end-diastolic (EDV), and end-systolic (ESV) volumes were calculated using a half-ellipsoid model of the LV (Ferry et al., 2015). From these volumes, LV ejection fraction (LVEF) was calculated using the formula: (EDV-ESV)/EDV × 100. The LV thickness / LV radius ratio (h/r) was also assessed in all animals. Echocardiographic measures were performed before (baseline) and 40 days (sacrifice) after tamoxifen injections (Ferry et al., 2015).

### Cardiac fibrosis

After sacrifice, hearts were cut in two equal halves following a line perpendicular to the short axis. Apical halves were then immediately fixed in Tissue-Tek (Sakura, USA) and frozen at −150°C in liquid nitrogen-cooled isopentane, and stored at −80°C until they were sliced into 8-μm-thick cryosections using an ultramicrotome (CM1860 Cryostat, Leica). Cardiac fibrosis was characterized with the PricroSirius Red staining. Frozen sections were fixed for 10 min in 3.7% formaldehyde, and washed with distilled water and 100% ethanol for 5 min. Sections were then transferred for 60 min in a 0.3% picric acid Sirius red solution, rinsed and fixed with acetic acid 0.5% for 10 min. After these steps, sections were dehydrated in a series of alcohols and then xylene and mounted in Eukitt. The extent of fibrosis was quantified using ImageJ software (12 randomly selected images per sample) and was expressed as the fibrotic content.

### Relative quantification of gene expression by qPCR

Total RNA was extracted from the apex of hearts using TRIzol® lysis reagent (Thermo Fisher Scientific, Saint-Herblain, France) and a tissue homogenizer (Bio-Gen PRO200) following the manufacturer's instructions. Extracted RNA was quantified by spectrophotometry using NanoDrop 2000 (Thermo Fisher Scientific, Saint-Herblain, France). From 500 ng of extracted RNA, first-strand cDNA was then synthesized using the high capacity cDNA reverse transcription kit (Thermo Fisher Scientific, Saint-Herblain, France) with random primers according to manufacturer's instructions. Using the Light Cycler® 480 system (Roche Diagnostics, Meylan, France), the reaction was carried out in duplicate for each sample in a 6-μl reaction volume containing 3 μl of SYBR Green Master Mix, 500 nM of the forward and reverse primers each and 3 μl of diluted (1:25) cDNA. The thermal profile for SYBR Green qPCR was 95°C for 8 min, followed by 40 cycles at 95°C for 15 s, 60°C for 15 s, and 72°C for 30 s. To exclude PCR products amplified from genomic DNA, primers were designed, when possible, to span one exon-exon junction. Data were collected and analyzed using the LightCycler® 480 software, release version 1.5.0 (Roche). Primers sequences used in this study are available on request. The gene expression stability of *Hmbs* (Hydroxymethylbilane synthase) was used as the reference transcript.

### Western blotting

Total proteins were extracted from the heart into an ice-cold lysis buffer containing Tris-HCl 20 mM at pH7.6, NaCl 250 mM, EDTA 3 mM, EGTA 3 mM, NP40 0.5%, DTT 2 mM, Na Orthovanadate 10 mM, Glycerophosphate 10 mM, and 2% of protease inhibitor cocktail (Sigma-Aldrich, Saint-Quentin Fallavier, France) and quantified with Bradford colorimetric assay. After a denaturation step for 5 min at 95°C, equal amounts of protein extracts (25 μg) were separated by SDS-PAGE before electrophoretic transfer onto a nitrocellulose membrane (GE Healthcare, Velizy-Villacoublay, France).

Western blot analysis was carried out using anti-Peroxisome proliferator-activated receptor gamma coactivator 1α (PGC1α (1:200, rabbit polyclonal, Santa Cruz Biotechnology, Heidelberg, Germany), anti-Voltage-dependent anion-selective channel 1 (VDAC1) (1:1000, mouse monoclonal, Abcam, Paris, France), anti-mitochondrial aconitase (ACO2) (1:2000, rabbit polyclonal, kindly provided by Pr Bertrand Friguet, University Pierre and Marie Curie), and an anti-Glyceraldehyde 3-phosphate dehydrogenase (GAPDH) (1:3000, rabbit polyclonal, Sigma-Aldrich, Saint-Quentin Fallavier, France). Proteins bound to primary antibodies were visualized with horseradish peroxidase (HRP)-conjugated secondary antibodies (Thermo-Fisher Scientific, Saint-Herblain, France) and a chemiluminescent detection system (ECL-Plus, GE Healthcare, Velizy-Villacoublay, France). Bands were quantified by densitometric software (Multi Gauge, Fujifilm).

### Statistical analysis

For echocardiography analysis, two-way ANOVA with repeated measures on one factor was performed. When an effect was detected, *post-hoc* Bonferroni comparisons were performed. For qPCR and western blots analysis, group normality and homogeneity of variances between groups were assessed using Shapiro-Wilk and Bartlett tests, respectively. Differences between SRF-HKO and SRF-HKO wheel groups were then statistically compared using *t*-test, or Kruskal & Wallis tests when normality or homogeneity of variance is not achieved. Values are expressed as mean ± S.E.M.

## Results

### Effects of voluntary exercise on cardiac function

Seven days after tamoxifen injection to induce SRF inactivation, twenty αMHC-MerCreMer:Sf/Sf mice were assigned to the sedentary (SRF-HKO, *n* = 8) and exercise (SRF-HKO wheel, *n* = 12) groups. The exercise group (SRF-HKO wheel) underwent 4 weeks of voluntary running on the wheel and daily running distance was recorded during 4 weeks. The longest daily running distance performed by SRF-HKO wheel mice has been reached during the third week of the voluntary training protocol (2.24 ± 0.25 km). The mean daily running distance was 1.8 ± 0.12 km/day. Moreover, the mean weight of mice before exercise protocol is 29.3 ± 1.4 g (baseline) and it did not differ between two groups after 4 weeks (sacrifice) (30.7 ± 2.7 SRF-HKO vs. 30.3 ± 1.7 SRF-HKO wheel). Voluntary exercise was initiated 7 days following the first tamoxifen injection, coinciding with a strong decrease in SRF mRNA and protein levels (Parlakian et al., 2005).

**Figure 1:**
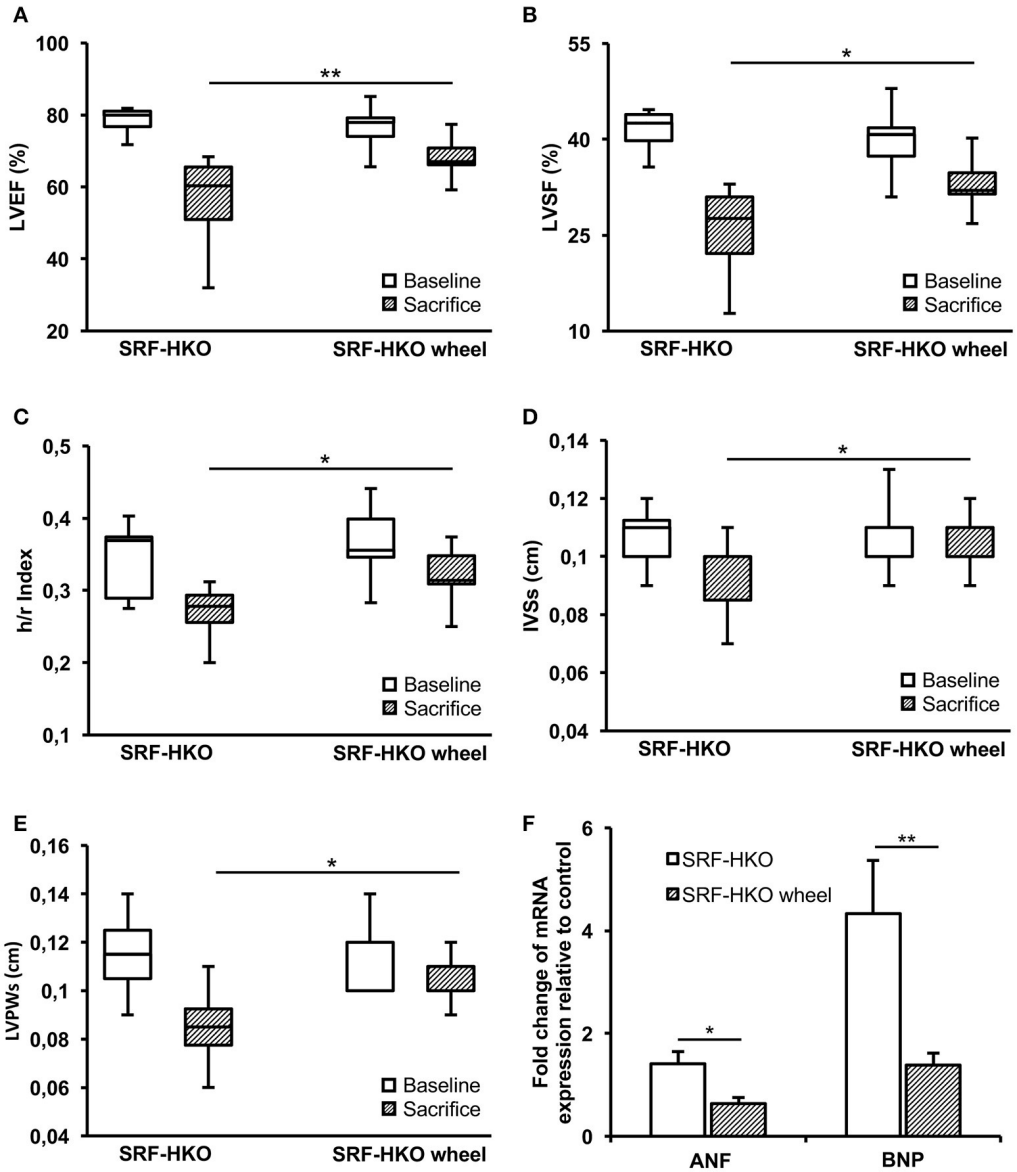
Left ventricle function and structure assessments by echocardiography **(A–E)** and real-time PCR **(F)** in SRF-HKO and SRF-HKO wheel mice. **(A–E)** Evolution of left ventricle ejection fraction (LVEF), left ventricle shortening fraction (LVSF), thickness/radius ratio (h/r), end-systolic inter ventricular septum thickness (IVSs) and end-systolic left ventricle posterior wall thickness (LVPWs) before (baseline) and 40 days (sacrifice) after tamoxifen injections in SRF-HKO (*n* = 8) and SRF-HKO wheel (*n* = 9) mice. **(F)** Relative quantification by real-time PCR of Atrial Natriuretic Factor (ANF) and Brain Natriuretic Peptide (BNP) mRNA levels in myocardial tissue. Results are presented in-terms of a fold change to control after normalizing with *Hmbs* mRNA. The control mice value is set at 1.0. Results are expressed as mean values ± SEM. ^*^p < 0.05; ^**^p < 0.01.

Before (baseline) and 4 weeks after voluntary activity (sacrifice), the LV function was assessed by echocardiography (Figure 1). We observed a significant decrease of LVEF between the baseline and the time of sacrifice both in SRF-HKO (78.4 ± 1.44 to 56.5 ± 4.32%, *p* < 0.01, Figure 1A) and in SRF-HKO wheel mice (76.8 ± 1.86 to 67.8 ± 1.77%, *p* < 0.01, Figure 1A) but this decrease was significantly lower in SRF-HKO wheel mice compared to SRF-HKO group (−9.0 ± 2.3 vs. −21.8 ± 3.5, *p* < 0.01). Similar results were observed for LVSF (−7.3 ± 5.7 vs. −15.6 ± 5.3%, *p* < 0.05, Figure 1B). We also demonstrated that LV h/r ratio was significantly greater in SRF-HKO wheel mice compared to sedentary SRF-HKO mice at the time of sacrifice (0.32 vs. 0.27, *p* < 0.05, Figure 1C) indicating that exercise limits the process of LV chamber dilatation. The sedentary SRF-HKO animals exhibited a significant decrease of IVSs at the time of sacrifice compared to baseline (−0.02 cm, *p* < 0.05) whereas this parameter did not change in SRF-HKO wheel (Figure 1D).

The IVSs thickness was maintained over the 4-week period in SRF-HKO wheel mice, but was thinner in sedentary SRF-HKO mice at the time of sacrifice (0.11 ± 0.01 vs. 0.09 ± 0.01, *p* < 0.05, Figure 1D). Both SRF-HKO and SRF-HKO wheel groups exhibited a significant decrease of LVPWs at the time of sacrifice compared to baseline (−0.03 ± 0.005 cm, *p* < 0.001, −0.009 ± 0.003, *p* < 0.01, respectively, Figure 1E). The thinning was significantly less pronounced in SRF-HKO wheel mice compared to sedentary SRF-HKO mice at the time of sacrifice (−0.02 cm, *p* < 0.01, Figure 1E). No significant differences were found during diastole between SRF-HKO wheel and SRF-HKO sedentary mice neither at baseline nor at the time of sacrifice. The LVEDD was significantly increased in SRF-HKO mice (+0.06 cm, *p* < 0.05) and the LVPWd was significantly decreased in SRF-HKO wheel mice (−0.01 cm, *p* < 0.05) between baseline and the time of sacrifice. The functional efficiency of voluntary physical activity on cardiac function during the establishment of HF was further reinforced by a reduction in the expression of the stress-induced atrial natriuretic factor (*p* < 0.05) and brain natriuretic peptide (*p* < 0.01) in exercised SRF-HKO wheel group compared to sedentary SRF-HKO mice (Figure 1F).

### Effects of voluntary exercise on cardiac fibrosis

**Figure 2:**
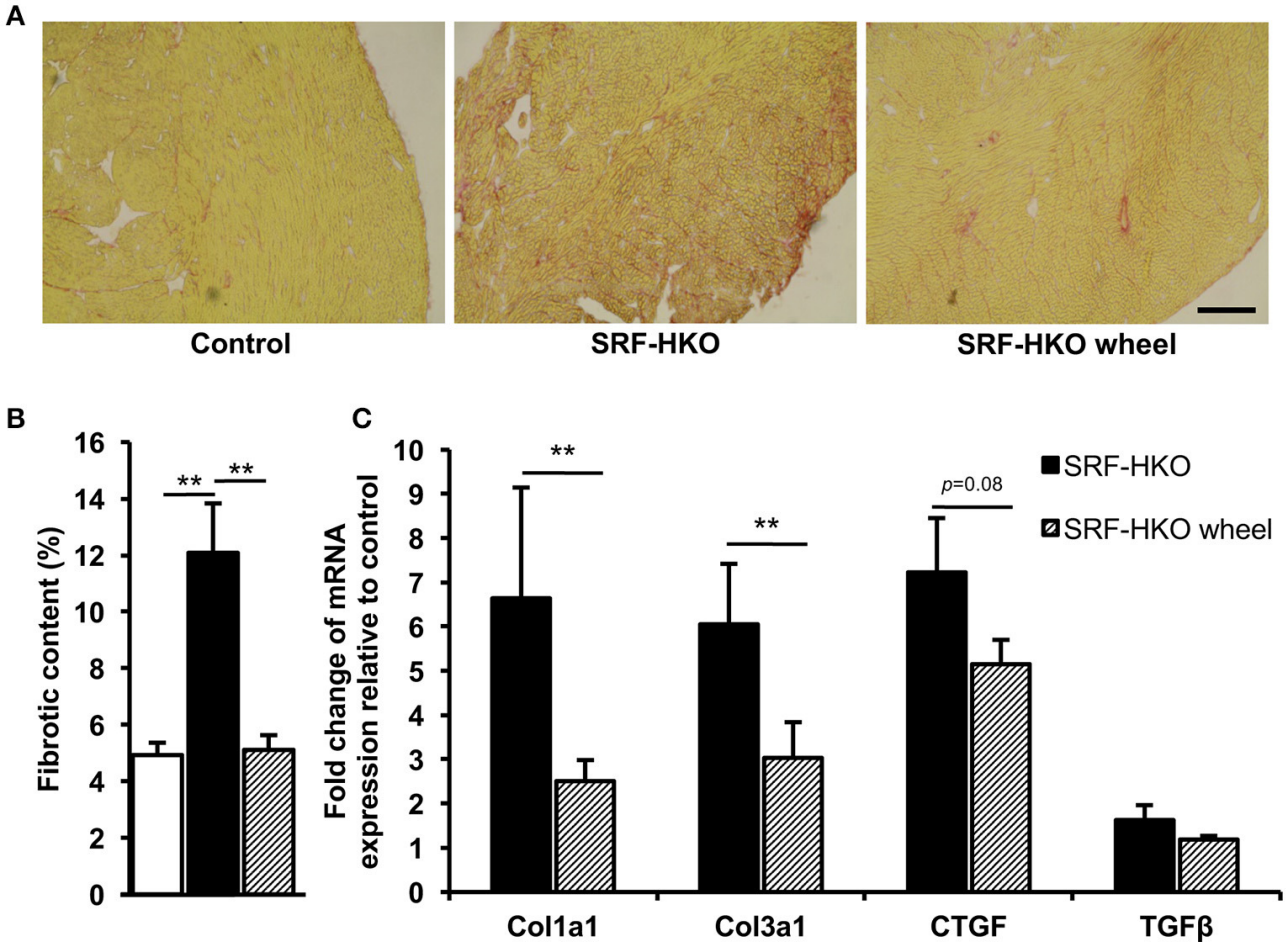
Myocardial fibrosis quantification by PicroSirius Red staining **(A,B)** and real-time PCR analysis **(C). (A,B)** PicroSirius Red staining of transverse myocardium sections **(A)** and histogram representation **(B)** of fibrotic content in control (*n* = 3), SRF-HKO (*n* = 5) and SRF-HKO wheel (*n* = 6) mice. **(C)** Relative quantification by real-time PCR of collagen I (Col1a1), collagen III (Col3a1), connective tissue growth factor (CTGF) and transforming growth factor beta (TGFβ) mRNA levels in myocardial tissue. Results are presented in-terms of a fold change to control after normalizing with *Hmbs* mRNA. The control mice value is set at 1.0. Results are expressed as mean values ± SEM. ^**^p < 0.01. Scale bar = 200 μm.

To evaluate the impact of voluntary exercise on cardiac remodeling, we analyzed myocardial fibrosis. Fibrosis were first measured by Sirius red staining. At the time of sacrifice, all of SRF-HKO mice exhibited interstitial fibrosis compared to control mice (12.0 vs. 4.9%, *p* < 0.01, Figures 2A,B). Nevertheless, the mean percentage area of fibrosis was lower in SRF-HKO wheel mice compared to SRF-HKO one (5.1 vs. 12.1%, *p* < 0.01). In addition, no difference was observed between SRF-HKO wheel and control mice (5.1 vs. 4.9%, *p* > 0.05).

Reduced fibrosis in the SRF-HKO wheel group in comparison to SRF-HKO groups was also supported by real-time PCR which demonstrated significant less induction of the expression of collagen I and III (Figure 2C). As showed in Figure 2C, both transforming growth factor beta 1 and connective tissue growth factor mRNA levels were also decreased in SRF-HKO wheel group but the differences failed to reach a significant level.

### Mitochondrial biogenesis and energetic pathways modifications of SRF-HKO mice

**Figure 3:**
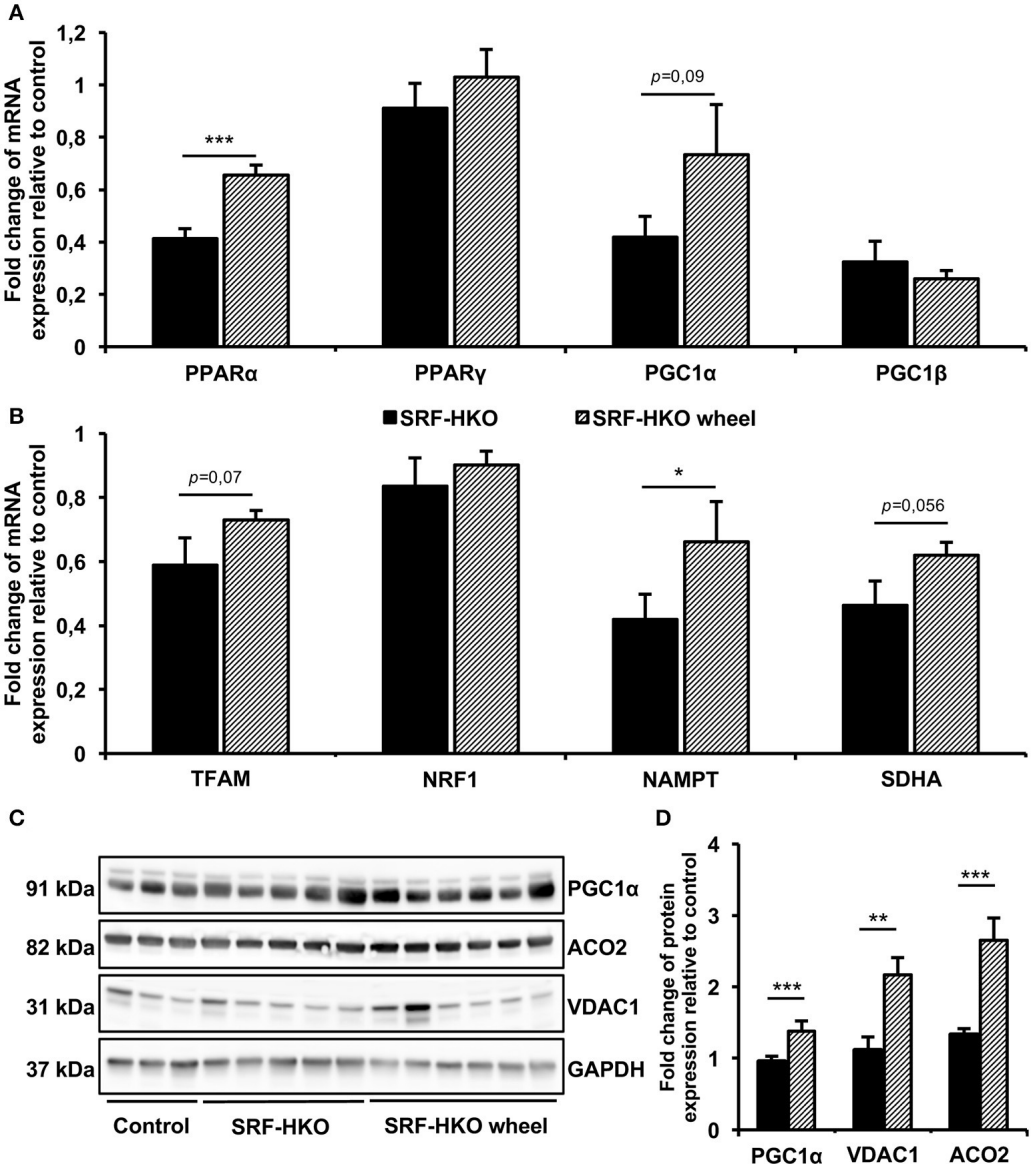
Real-time PCR **(A,B)** and Western blot analysis **(C,D)** of mitochondrial biogenesis and selected energetic pathways. **(A,B)** Relative quantification by real-time PCR of peroxysome proliferator-activated receptors alpha and gamma (PPARα/γ), PPAR gamma coactivators-1 alpha and beta (PGC1α/β), Transcription factor of activated mitochondria (TFAM), nuclear respiratory factor 1 (NRF1), nicotinamide phosphoribosyltransferase (NAMPT) and succinate deshydrogenase complex subunit (SDHA) mRNA levels in myocardial tissue. Results are presented in-terms of a fold change to control after normalizing with *Hmbs* mRNA. The control mice value is set at 1.0. **(C,D)** Western blot analysis **(C)** and histogram representation **(D)** of PGC1α, mitochondrial Aconitase (ACO2) and Voltage-Dependent Anion-selective Channel 1 (VDAC1) proteins quantification. Results are presented as a fold change to control after normalizing with GAPDH protein. The control mice value is set at 1.0. Results are expressed as mean values ± SEM. ^*^p < 0.05, ^**^p < 0.01, ^***^p < 0.001.

To gain a mechanistic insight on the beneficial effects of voluntary exercise, we examined the mitochondrial biogenesis and energetic pathways markers expression profile at mRNA and protein levels (Figure 3). The real-time PCR showed that PPARα gene expression was significantly higher in sedentary SRF-HKO wheel mice compared to sedentary SRF-HKO mice at the time of sacrifice (*p* < 0.001, Figure 3A). Moreover, the PGC1α gene expression tended to be greater in SRF-HKO wheel animals compared to their sedentary counterparts without reaching statistical significance (*p* = 0.09, Figure 3A). The PPARγ and PGC1ß genes expression remained unchanged between the two SRF-HKO groups at the time of sacrifice (Figure 3A). Concerning other mitochondrial markers, the expression of NAMPT gene, which is involved in NAD biosynthesis from nicotinamide, was significantly greater in SRF-HKO wheel mice compared to SRF-HKO mice at the time of sacrifice (*p* < 0.05, Figure 3B). The TFAM (*p* = 0.07) and SDHA (*p* = 0.056) genes expression, implicated in metabolic adaptation expression, tended to be greater in SRF-HKO wheel animals compared to SRF-HKO mice but these differences did not reach statistical significance (Figure 3B). The NRF1 gene expression remained unchanged between the two SRF-HKO groups at the time of sacrifice (Figure 3B).

Modifications in mitochondrial biogenesis and energetic pathways of SRF-HKO mice after voluntary exercise training were also supported by western-blot analysis (Figures 3C,D), which demonstrated that PGC1α (*p* < 0.001), VDAC1 (*p* < 0.01), and ACO2 (*p* < 0.001) proteins levels were significantly increased in SRF-HKO wheel group compared to the sedentary group (Figures 3C,D).

## Discussion

The major finding of this study is that a 4-week voluntary exercise stabilizes the functional outcomes of non-ischemic dilated mouse hearts and this benefit is accompanied with a reduction of myocardial fibrosis and the enhancement of mitochondrial energy metabolism.

Non-ischemic DCM is one of the most frequent diseases leading to HF in young adult patients (Savvatis et al., 2015). It is characterized by ventricular dilation, altered systolic function and in some cases diastolic dysfunction. In contrast to ischemic cardiomyopathies which are amenable to a variety of pharmacological, electrical, and interventional treatments, the only treatment of advanced stage of non-ischemic form of HF is heart transplantation or permanent implantation of an assist device (Clegg et al., 2006; Hamdi et al., 2013). Thus, there is a real need to develop novel therapies for these cardiomyopathies. In the present study, we reported a significant beneficial impact of 4-week voluntary running protocol on LV function and remodeling in a mouse model of DCM induced by a cardiac-specific inactivation of SRF. We demonstrated that voluntary exercise can preserve the contractile function and reduce the extent of ventricular dilation of SRF-deficient mice. We also confirmed that voluntary exercise could stabilize the systolic function in SRF-deficient transgenic mice, although the ventricular dilatation in diastole still takes place. The lack of impact of exercise on the LV chamber diameter in diastole may not be surprising when one considers that endurance training is associated with LV hypertrophy, but also chamber dilatation, as an adaptive remodeling to increased body perfusion demand (King and Wood, 2013).

SRF is a transcription factor of the MADS-box family, and takes part in the regulation of the genes expression involved in many cellular processes such as cellular growth, proliferation, differentiation, the actin remodeling. An abnormal truncated form of SRF has been described in human failing hearts (Davis et al., 2002) and the cleavage of SRF by caspase has been found to potentially promote HF (Chang et al., 2003). Conditional invalidation of SRF in cardiomyocytes demonstrates that SRF is essential for cardiac maturation during embryogenesis and the postnatal period (Parlakian et al., 2005; Gary-Bobo et al., 2008). In this study, we used conditionally invalidated SRF mice (αMHC-MerCreMer:Sf/Sf) which allows to inactivate SRF gene expression specifically in cardiomyocytes since tamoxifen-inducible Cre recombinase expression is under the control of the cardiomyocyte-specific alpha myosin heavy chain promoter (Parlakian et al., 2005). There is a important decrease in SRF both in mRNA and protein levels as early as 5 days after tamoxifen; mice then progressively exhibit a decline of cardiac function, associated with reduced metabolic flux to the myofibril and decrease contractility, leading to dilated cardiomyopathy, HF, and death within 10 weeks (Parlakian et al., 2005). All these observations underline that αMHC-MerCreMer:Sf/Sf mice a good model to evaluate the impact of voluntary physical activity during the establishment of HF in a non-ischemic cardiomyopathy context.

It is now widely accepted that chronic physical activity is beneficial for cardiac health and particularly on HF (Schocken et al., 2008; Shiroma and Lee, 2010). Few data are available regarding the impact of exercise on cardiac function and structure in the context of DCM. It has been demonstrated that an 8-week cycle exercise program (Holloway et al., 2012) or a 12-week cardiac rehabilitation program (Legallois et al., 2016) improved cardiac function of patients with HF from DCM. Other studies demonstrated that exercise is associated with a decrease in plasma concentration of C-reactive protein (CRP), TNF-α, and Serum amyloid A (SAA) in patients suffering from chronic HF (Larsen et al., 2001; Gill and Malkova, 2006; Batista et al., 2009). The exact mechanisms underlying beneficial and anti-inflammatory effects of physical activity are not clearly understood. More recently, Bei et al. (2017) demonstrated that exercise induced an increase of circulating extracellular vesicles in mice. Exercise-derived extracellular vesicles can confer the systemic benefits of exercise to distal organs, including the heart. Briefly, the authors demonstrated that exercise induced an increase in circulating extracellular vesicles, *via* the activation of the ERK1/2 and HSP27 signaling, thus providing an enhanced protection against cardiac injury compared to endogenous extracellular vesicles at baseline. This study suggested that the extracellular vesicles might serve as a potent therapy for myocardial injury in the future (Bei et al., 2017).

Three types of chronic exercise dominate the literature (i) forced treadmill running, (ii) swimming, and (iii) voluntary wheel running. A majority of studies have used a motorized treadmill that varied in intensity, slope and duration between the studies. In addition, both treadmill and swimming are known to induce stress. Despite its non-forced nature, voluntary wheel-running is sufficient signals to induce physiologic cardiac growth in rodents (Allen et al., 2001; Natali et al., 2001; Konhilas et al., 2004). Similar to humans and forced treadmill running, a minimum of 3–4 weeks of exercise is required to instigate a 5–20% increase in cardiac mass depending on strain, sex, diet, age, or any combination of these variables (Allen et al., 2001; Lerman et al., 2002; Konhilas et al., 2004, 2015; McMullan et al., 2016). We therefore employed a 4-week non-forced and non-stressful voluntary wheel-running protocol to provide sufficient stimuli for cardiac adaptation. Our first results demonstrated that the mean daily running distance of our DCM mice is relatively modest compare to mouse capacities in forced treadmill running but enough to improve the cardiac function, reduce the LV cavity dilation, prevent the myocardial fibrotic content accumulation and reduce the cardiac stress in SRF-HKO wheel mice compared to their sedentary counterparts. Moreover, the increased PPARγ gene expression and the increased PGC1α, VDAC1, and ACO2 proteins expression found in the SRF-HKO wheel mice show that a 4-week voluntary exercise was already sufficient to induce mitochondrial function improvement and biogenesis. All in one, these latter results suggest that voluntary exercise is beneficial and mitigates or prevents the development of DCM induced by a specific inactivation of SRF into the myocardium.

Molecular processes leading to DCM and lately HF can be very heterogeneous depending on the subject and the stage of the disease. In this regard, and because the heart is one of the highly oxidative organs, many studies explored the impact of mitochondrial dysfunction in the development of cardiomyopathies (El-Hattab and Scaglia, 2016). Interestingly, it has been shown that DCM is one of the most common types of cardiomyopathies induced by mitochondrial disorders (Bates et al., 2012; Finsterer and Kothari, 2014). However, to our knowledge, no data is available regarding the potential implication of SRF inactivation-induced mitochondrial disorders in the development of DCM. In the present study, we showed that the inactivation of SRF in αMHC-MerCreMer:Sf/Sf mice lead to a significant decrease of the myocardial genetic expression of PPARγ, PGC1α, TFAM, NAMPT, and SDHA compared to the control mice. These results demonstrated that cardiac specific SRF inactivation affect mitochondrial biogenesis, transcription activity and nicotinamide biosynthetic enzyme activity which represent potential underlying mechanisms involved in the development of DCM. Moreover, our results suggested for the first time that 4-week voluntary exercise might represent a beneficial way to prevent the mitochondrial disorders potentially involved in cardiac dysfunction in the context of non-ischemic DCM. Thus, our finding can help define new molecular and cellular triggers that may initiate the DCM and represent new therapeutic targets.

In conclusion, our murine model of DCM is a good model to evaluate the impact of voluntary physical activity during the establishment of HF in a non-ischemic cardiomyopathy context. Indeed, 4 weeks of voluntary running induce significant positive impact on cardiac function despite the use of 9 old-months mice which could be potential confounding factor for physiological adaptation.

## Author contributions

OA, AF, conceived the study. RD, DV, PN, ZL, MM, AF, and OA, performed the laboratory experiments. NM performed functional assessement. RD and MM contributed to the statistical analysis. RD, DV, and OA wrote the draft of the paper. RD, DV, ZL, MM, AF, and OA, reviewed the manuscript. All authors have read and approved the final manuscript.

### Conflict of interest statement

The authors declare that the research was conducted in the absence of any commercial or financial relationships that could be construed as a potential conflict of interest.
